# Healthcare in Africa

**Published:** 2009-10

**Authors:** V Grigorov

**Affiliations:** ARWYP Medical Centre, Johannesburg

## Prior to the recession in the USA

In 2006, just before the economic turmoil, United States hospitals provided $28.8 billion dollars worth of care for patients who had no form of medical insurance (uncompensated care).^1^ This is twice the amount proposed for the entire budget (R100 billion) of the South African National Health Insurance (NHI) scheme.

The burden was heavier on certain hospitals than others. What they call in America a ‘safety-net’ hospital is one that provides in-patient care to a greater proportion of uninsured patients. Only 2% of the hospitals in America are members of the National Association of Public Hospitals and Health Systems (NAPH), and they have to care for about 25% of the uncompensated in-patient population. The safety-net hospitals are located in areas where many uninsured patients live. The hospitals recoup the cost of care by charging the insured population higher fees. Despite that, most safety-net hospitals have negative margins.^2^,^3^

Safety-net hospitals in America serve as educational centres as well as disaster recovery centres. (America does not have ‘government’ hospitals. They are all private in one sense or another, as they are either self-funded, funded by research, or receive additional help from the State through education.)

## Medical insurance in the USA

Medicare is a social security tax levied on every working individual in the United States until they reach retirement age. Some of the tax goes towards the funding of retirement medical care. However, this fund (at current rates) will be bankrupt in about 15 years.

Medicaid is a form of subsidy that the Federal Government gives to families with low incomes and no assets; i.e. below $800 US a month. The individual states receive their medical funding from the Federal Government. Some time ago the individual states were allowed to decouple their payments between the two programmes. The states could then pay separately for the Medicare and Medicaid patient.

Congress (i.e. the Federal Government) suggested that those states that had more safety-net hospitals should provide more funding of Medicaid and Medicare to the hospitals. The individual states did not adopt this system in the 1980s. In the 1990s, all hospitals could claim directly from the Federal Government, but through the State. As a result, the State got the lion’s share of the money through the taxes.

At the same time, the hospitals and the individual states understood that they could tap more funds from the Federal Government through the so-called process of ‘recycling’. In this scenario, the more they claimed, the more they received, but the surplus went as a tax to the individual state’s coffers. What is more important is that recycling was difficult to stop. Even when the Clinton administration tried to clamp down by decreasing the Medicaid payments by 5%, the practice still continued. The effect at that time was a squeeze on all the doctors in the hospitals to receive less payment.

However, the practice of recycling was stopped in the individual states after an inquiry. The various states currently have differing levels of Federal financing – ranging from $200 000 in Wyoming to $1.5 billion in New York. Different hospitals have different levels of Federal healthcare providers. So some hospitals can claim more than others, irrespective of the care provided.

This highlights only some of the problems of the USA healthcare system.^4^ One can therefore get an idea of the magnitude of the challenges facing the American healthcare system from this and also from what is reported daily in the media. Even in a society as complex as America, the state-provided care is riddled with some kind of fraud or profiteering at State level or by the providers.

Their society is highly sophisticated, with excellent electronic monitoring systems as well as electronic patients’ records. These include all the Veteran Administration hospitals as well as most of the university and private centres. (The Veteran Administration system is only for soldiers and officers who fought in wars for the United States.) Due to confidentiality issues, patients’ records cannot be accessed at a federal level; however, patients take their electronic records with them when they move.

## Africa

A recent article in the McKinsey Quarterly of March 2008 reviewed the options of healthcare in Africa. Africa (including southern Africa) is mostly an agrarian society.^5^ As Moeletsi Mbeki states in his book, *Architects of Poverty*, ‘…the great majority of Africans are today experiencing the opposite – less security and comfort. In fact, in many instances they face hunger, homelessness, the threat of violence, actual violence, disease and starvation’.^6^

Further in his book he states that, ‘…sub-Saharan Africa today consists of fossilised pre-industrial and pre-agrarian-revolution social formations, and therein lies their inability to grow economically. The absence of an industrial revolution on the African subcontinent has left it with socio-economic structures that are, in the main, degenerative rather than accumulative’.^6^

Irrespective of trumpeting otherwise in the press, inequality in Africa has risen significantly in the last 30 years. The number of Nigerians living below the poverty line has increased from 19 million in 1970 to 90 million in 2000. In 1970, the top 2% of the population earned the same as the bottom 17%. By 2000 the income of the top 2% was equal to the bottom 55%.^7^

As Mbeki says, to understand the difference is to compare Nigeria with China. The GDP of Nigeria halved for the period that China managed to lift 400 million above the poverty line. Just as a matter of interest, Equatorial Guinea pumps more oil per capita than Saudi Arabia, and compare the difference in poverty in those two countries.^6^

Currently, South Africa has a new ruling class that decides what ‘transformation’ is. More importantly, it is the new ruling class that decides what is non-desirable transformation. The impact of the trade unions (Cosatu) has been marginal, despite their strenuous efforts to change South Africa’s economic and social policies.^6^

From the time of Karl Marx, it has been observed that the dominant ideas of a particular society are the ideas of the dominant classes. South Africa is dominated not by one class, but by two. The black upper class dominates the country’s political life but has no role in the ownership. Its main role is to oversee the redistribution of wealth towards consumption, and manages (mismanages?) a few state-owned enterprises inherited from the National Party era.^6^

With globalisation and the importation of cheap goods, the so-called mineral energy complex (MEC) (controlling most of the activities in the country) has managed to destroy the non-MEC manufacturing industries. This has led to a massive increase in unemployment.^6^

## An economist’s view of sub-Saharan Africa’s provision of healthcare

Research from the Bill and Melinda Gates Foundation has estimated that the growth in demand for private healthcare in sub-Saharan Africa could add an additional $20 billion per year investment in private healthcare.

The McKinsey Quarterly of March 2008 pointed out that in 2005, of the total health expenditure in Africa of $16.7 billion, 60% was privately financed, and half of that exppenditure went to private providers. Furthermore, they found that the private sector does not primarily serve the wealthy. In Ethiopia, Nigeria, Kenya and Uganda, the World Bank found that more than 40% of the people in the lowest economic quintile received healthcare from private, for-profit providers. The private-sector role in countries like Uganda and Ghana is 60%, in Namibia it is 10%.^5^

The authors of this report predict that the private healthcare expenditure in sub-Saharan Africa will double by 2016 to $35 billion per year. This equates to a current value of R300 billion, or nearly half of the GDP of South Africa.^5^ Furthermore, they speculate that most of the medical projects will be small – in the region of under $3 million. They will involve the areas of life sciences, distribution, retailing, risk pooling and medical education. Most of the capacity expansion will be in the area of hospital beds and refurbishing existing assets.^5^

In research conducted in Tanzania, it was noted that generally, the public prefers private rather than public providers (75% of the patients), citing convenience and quality of care among other benefits.^5^ Private institutions are better supplied with medicines than their government counterparts.5 At the same time, it should be admitted that private business fights an ongoing battle regarding unethical business practices, counterfeiting and inconsistent care.

It is thought that public–private collaboration would expand the risk-pooling arrangements. Currently, both sides are highly suspicious of each other. The private providers see the Government’s failures every day, in all spheres of delivery, especially in healthcare. Government sees the private providers as ‘money hungry’.

The Constitution^8^ is often quoted by the Government regarding the ‘right to care’ and therefore the need for an NHI. According to Chapter 27, regarding healthcare, food, water and social security:

1. ‘Everyone has the right to have access to:
(a) healthcare services, including reproductive healthcare;
(b) sufficient food and water; and
(c) social security, including, if they are unable to support themselves and their dependants, appropriate social assistance.
2. The State must take reasonable legislative and other measures, within its available resources, to achieve the progressive realisation of each of these rights.
3. No one may be refused emergency medical treatment.’

Everyone agrees that paragraph 3 is enacted in all the private hospitals, but not the public hospitals.^9^ One can see that the provisions cited by the African National Congress for constitutional rights are not as they appear to be. The Constitution notes that the State ‘in its resources’ must take ‘reasonable measures’. Of course this depends on what the ruling party understands by reasonable.^8^

When one reviews the abundance of literature available by economists in this field, one realises that the option of centralised healthcare is not viable, and is in fact dangerous because the constitutional right to healthcare would be violated in the process of migration to public care, should this system be implemented. The term ‘public care’ is not even mentioned in the Constitution.

It is argued that South Africa will need to spend at least 5% of the GDP on healthcare.^10^ However, even if that does occur, the ‘package’ will purchase even less than the lowest tier of the most basic current medical scheme. This is irrespective of the cost of employment at the present stage. The preference for individuals to utilise additional (extra cost) private providers (the latter are assumed not to be abolished) is going to be an additional burden.

Dr Broomberg, in a recent article, points out that currently the medical fraternity is believed to be significantly underpaid compared with counterparts overseas.^10^ This may be true, as currently a medical student in South Africa spends approximately R1 million on education, lodging, books, etc. In the United States the current course of four years’ education is $30 000 per year – which is approximately $120 000–150 000 for the total period of study. The pay of a junior state-employed doctor in South Africa is in the range of $12 000 per year (before overtime), whereas the equivalent United States doctor starts at a minimum of $100 000 per year (after residency/registrarship).

Private-sector users are accustomed to quality care. The inclusion of this group in an ineffective enterprise (funded by the taxpayer base in South Africa) would be dangerous for the economic stability in the short and long term. Even if it is argued that repressive measures regarding possible refusal of tax collections can be used, in the long term there will be definitive repercussions for the economy linked to emigration. (Compared with the lowest classes whose main objective is food stability, the middle class’s main objective is health and education.)

There is an argument regarding doctors’ rights of income. The different specialist forums argue that their income would be significantly decreased by the introduction of the NHI. It is often argued that the doctors’ rights are infringed upon with the decision of the Government to abolish private practice. This is not true. The Constitution states in Chapter 22, Freedom of trade, occupation and profession: ‘Every citizen has the right to choose their trade, occupation or profession freely. The practice of a trade, occupation or profession may be regulated by law’. This clearly states that the Government can abolish or regulate the use of private practice if it deems it necessary to regulate it by law.

Of course it is completely another question regarding the controversy surrounding the previous section of the Constitution mentioned, i.e. Chapter 27, as well the economic viability of the plan. If one looks at it pragmatically, an additional income for the South African Government of more than $20 billion dollars (R100–160 billion) is more than welcome as it definitely would not spend that amount of money on healthcare (see Federal income and State expenditure already cited).

If one looks back at the history of what was spent in the apartheid era on research, the Medical Research Council budget in 1986–1987 was approximately R100 million,^11^ which at that time equated to approximately $50 million (adjusted for inflation to $150 million today). Minister Mangena stated in the Science and Technology Departmental budget vote for 2007/2008^12^ that it allocated R200 million to the ‘South African Research Chair’s Initiative’. This is in current terms in the range of $20 million (although there is no relationship between the two).

It is also interesting to do a simple mathematical calculation, from the supply side of the economy. Currently in South Africa, there are 184 registered cardiologists (adult) and 30 paediatric cardiologists. Over the last seven years, 40 cardiologists have qualified (5.7 per year). How many of those in practice currently are at retirement age is unknown, but what is known is the fact that more than 50% of the cardiologists are way above 45 years of age with most of them above 55.

If one assumes that in the best-case scenario the status quo will be kept, it will mean that for the current population of about 46 million, there will be only 220 cardiologists. Therefore each cardiologist will have to see in the range of 210 000 patients. As a comparison for the same size of population, Italy has approximately 5 000 cardiologists and it is deemed ‘unserved’.

## Conclusion

It is a strange phenomenon that in a time when the world’s population is living longer and rich countries are realising that State healthcare is consuming more public finances than they bargained for, South Africa wants to go in the opposite direction. The administration of Obama has a saying, ‘never waste a good crisis’, meaning that in difficult times, difficult decisions are easier to swallow.

What is America working on at the moment? Taxing workers’ employer-provided medical benefits could become the next big controversy for President Obama in his quest to overhaul the healthcare system of America. And the biggest opposition will come from the trade unions. It is thought that a $1-trillion, 10-year package can’t be paid for without taxing at least some of the high-value healthcare plans.

The most important thing for the USA president to do is not to increase the deficit. What is mooted by United States senators at the moment is to set a limit for family coverage and individuals (this limit is above the amount that the American Blue Shield charges for a medium-sized American family, i.e. $17 000 for a family and $6 800 for an individual per year). Plans above that amount will be taxed.^13^

America has already had an affair with the so-called ‘singlepayer’ plans. The major problem with that idea is that the payroll taxes will increase. America estimates that a single-payer system will mean an increase of 3.3% more in taxes.^14^

There is another snag. All subsidies for healthcare are expensive. At the same time, America (as well as South Africa) is attacking the symptoms but not the cause. In the United States, health insurance is tax deductible for corporations. That begs the question – what about those without such insurance who have to subsidise the insured via their taxes? They are also subsidising ‘gold-plated’ insurance schemes where the cost is not known.^15^

Planning ‘free healthcare for all’ is a nice idea, but let’s not forget that a budget of more than R100 billion per year will attract a lot of prying eyes for misappropriation – this equates to at least two ‘arms deals’ per year! And what about the doctors? The Romans said 2 000 years ago, ‘Vini Bene Vini Patria’.
